# Natural Genetic Variation in Yeast Reveals That NEDD4 Is a Conserved Modifier of Mutant Polyglutamine Aggregation

**DOI:** 10.1534/g3.118.200289

**Published:** 2018-09-11

**Authors:** Theodore W. Peters, Christopher S. Nelson, Akos A. Gerencser, Kathleen J. Dumas, Brandon Tavshanjian, Kyu Chul Chang, Gordon J. Lithgow, Robert E. Hughes

**Affiliations:** Buck Institute for Research on Aging, 8001 Redwood Blvd, Novato CA, 94945

**Keywords:** yeast, natural genetic variation, protein aggregation, polyglutamine, Huntington’s disease

## Abstract

A feature common to late onset proteinopathic disorders is an accumulation of toxic protein conformers and aggregates in affected tissues. In the search for potential drug targets, many studies used high-throughput screens to find genes that modify the cytotoxicity of misfolded proteins. A complement to this approach is to focus on strategies that use protein aggregation as a phenotypic readout to identify pathways that control aggregate formation and maintenance. Here we use natural variation between strains of budding yeast to genetically map loci that influence the aggregation of a polyglutamine-containing protein derived from a mutant form of huntingtin, the causative agent in Huntington disease. Linkage analysis of progeny derived from a cross between wild and laboratory yeast strains revealed two polymorphic loci that modify polyglutamine aggregation. One locus contains the gene *RFU1* which modifies ubiquitination states of misfolded proteins targeted by the E3-ubiquitin ligase complex Rsp5. Activity of the Rsp5 complex, and the mammalian homolog NEDD4, are critical in maintaining protein homeostasis in response to proteomic stress. Our analysis also showed linkage of the aggregation phenotype to a distinct locus containing a gene encoding the Rsp5-interacting Bul2 protein. Allele-swap experiments validated the impact of both *RFU1* and *BUL2* on huntingtin aggregation. Furthermore, we found that the nematode* Caenorhabditis elegans*’ ortholog of *Rsp5*, *wwp-1*, also negatively regulates polyglutamine aggregation. Knockdown of the NEDD4 in human cells likewise altered polyglutamine aggregation. Taken together, these results implicate conserved processes involving the ubiquitin regulation network that modify protein aggregation and provide novel therapeutic targets for polyglutamine and other protein folding diseases.

Defects in protein homeostasis are implicated in a number of late onset human diseases, including Alzheimer’s, Parkinson’s and Huntington’s disease (HD) (Balch *et al.* 2008; Douglas and Dillin 2010). HD is an inherited fatal neurodegenerative disease caused by a CAG expansion in the human HTT locus (1993; Saudou and Humbert 2016). Expression of expanded polyglutamine (polyQ) tracts in the huntingtin protein is the ultimate cause of HD. While it is known that age of onset in HD is negatively correlated with the length of CAG expansion, patients with identical HTT disease alleles can differ significantly in their age of onset (Gusella and Macdonald 2009; Paulson and Albin 2011). An estimated 40% of the remaining variability in age of onset in HD (after accounting for Q tract length) is due to genetic modifiers elsewhere in the genome (Genetic Modifiers Of Huntington’s Disease (Gem-Hd) Consortium 2015; Djoussé *et al.* 2003; Gayán *et al.* 2008; Wexler *et al.* 2004). A central goal in the field is to identify such modifier genes, to better understand pathogenic mechanisms and potentially identify novel drug targets.

A pathological hallmark of HD is the presence of neuronal aggregates of mutant huntingtin in specific regions of the brain that undergo degeneration (Difiglia *et al.* 1997). The propensity for CAG-expanded huntingtin to form aggregates in cells has been recapitulated in numerous transgenic systems including mouse and yeast (Hughes *et al.* 2001; Krobitsch and Lindquist 2000; Mangiarini *et al.* 1996). A wide range of experiments in cell-based and cell-free systems indicate that mutant huntingtin can spontaneously assemble into amyloid-like structures, nucleating from oligomers and fibrils, eventually forming microscopically visible aggregates (Trepte *et al.* 2014; Wetzel 2012). The precise role of aggregated forms of huntingtin in disease pathology remains a subject of debate, but intracellular aggregates are generally considered to be a sign of proteotoxic stress (Arrasate and Finkbeiner 2012). Given the need for novel insights into natural intracellular defenses against polyQ aggregate formation and toxicity in HD and other diseases, we sought to use natural genetic variation to identify genes that play a role in HTT aggregation. Natural-variation based mapping of protein aggregation determinants has been applied most notably in nematodes, and has identified candidate loci that modify huntingtin toxicity and aggregation, yet the specific genes regulating these traits remained elusive (Gidalevitz *et al.* 2013). These successes lent us confidence in our approach and we sought to extend the mapping of candidates into molecular validation of candidate aggregation effectors in multiple species.

In this work, we set out to develop a screening strategy for natural variants that modify the control of aggregation of an expanded polyQ huntingtin fragment in a model system enabling expedient validation of aggregation-linked loci. As budding yeast can be a powerful model for the discovery of conserved modifiers of mutant polyQ toxicity (Duennwald *et al.* 2006a; Giorgini *et al.* 2005; Kaiser *et al.* 2013; Kayatekin *et al.* 2014; Mason *et al.* 2013; Willingham *et al.* 2003), we chose yeast for our natural variation-based screen using protein aggregation as a quantitative phenotype. We used one candidate pathway emerging from our screen that of the ubiquitin ligase Rsp5/WWP-1/NEDD-4, as a basis for further characterization of modifiers in nematodes and human cells.

## Materials and Methods

### Yeast strains, media, and methods

Yeast strains used in this study are shown in Table S1. Cultures were grown at 30° in rich media with 2% glucose or synthetic complete media (0.67% yeast nitrogen base with ammonium sulfate, 2% glucose, and required amino acids) (Sherman 1991). Overnight cultures were refreshed 1:40 in appropriate media, grown for six hours and plated for image analysis. [*PIN*] status of parent strains was determined by aggregation of the RNQ1-YFP reporter protein expressed from the *CUP1* promoter (Addgene plasmid p15596 (Duennwald *et al.* 2006a; Liu and Lindquist 1999) after 24 hr of induction with 100 µM CuSO_4_. Strains were cured via five successive passages on rich media containing 5 mM GuHCl (Byrne *et al.* 2007). Cells were transformed with a p416GPD-derived plasmid expressing an HTT exon1 fragment fused to GFP using standard protocols (Hughes *et al.* 2001).

### Microscopy and quantitation of fluorescence

Microscopy of sonicated and freshly seeded yeast in 96-well microplates coated with 2 mg/ml concanavalin A (Sigma) was performed on a Nikon Eclipse Ti-perfect focusing system (PFS) inverted epifluorescence microscope. Microscope automation using NIS Elements 4.0 (Nikon) captured large, tiled field of views in each well of the microplate. Automated image analysis using Image Analyst MKII (Novato, CA) identified yeast cells as bright objects in slightly off-focus differential interference contrast (DIC) images. GFP fluorescence captured using a standard GFP filter set (Semrock) and quantitated using Image Analyst MKII. To define cells containing fluorescence and establish parameters to detect GFP foci representative of protein aggregates, high-pass spatial filtering was used to amplify fluorescent objects of interest and remove background signal (Gerencser *et al.* 2008). DIC and GFP images were segmented using a locally adaptive technique. Thresholds for sensitivity were automatically determined based on a positive control (a high-aggregating yeast image). A high-pass filter specific for small, punctate objects (<2 µm) was used to define aggregates; objects >2 µm in diameter were defined as diffuse fluorescence. This algorithm was iteratively optimized to maximize the distinction between fluorescence patterns and matched with results of visual scoring.

To determine the percentage of fluorescent cells with foci for a given strain background (segregants from the lab-vineyard cross, wild-type parents, or allele swaps), we tabulated the number of cells containing foci divided by the total number of fluorescent cells. Reported phenotypes for each strain are the average of at least five individual clones from the Htt-GFP transformation for each strain.

### Linkage analysis

Standard quantitative trait loci analysis was performed using the genotype data from (Brem and Kruglyak 2005). Considering our set of phenotyped segregants, we filtered out redundant polymorphic DNA sequence markers that had high genotype correlation (R^2^ > 0.90) to neighboring markers. Removing the first member of highly correlated marker pairs, blocks of correlated markers were filtered out to leave the closest pair of markers that passes the filter (*i.e.*, R^2^ ≤ 0.90). We also removed all markers with more than one missing genotype entry or less than 25% frequency of either allele. Each of 628 markers was considered for linkage to the phenotype of interest by defining the sets of haploid segregants harboring the laboratory and vineyard allele, respectively, and comparing phenotypes among the two sets with a linear regression test. The test reported a *p*-value for the hypothesis that the fitted slope between the phenotypes of the two allelic groups equaled zero. The false discovery rate at a given *p*-value threshold was estimated as the ratio of two quantities: the average number of markers exhibiting significance below the threshold across 100 sets of permuted data, and the number of true markers exhibiting significance below the threshold in real data.

### Statistical analysis

Student’s *t*-test was used to evaluate the effects of experimentally perturbing candidate genes in yeast experiments in Figure 4 and Figure 5. Error bars indicate standard error throughout. To test the significance of the wwp-1 RNAi on puncta per worm, we used a one-way ANOVA with Sidak’s multiple comparisons test, which returned *P* < 0.0001. Statistical significance was tested in Figure 6 using a Mann-Whitney rank sum test.

### RFU1 allele swap

The *RFU1* locus from the vineyard strain RM11-1a was introduced at the endogenous location into the haploid laboratory strain BY4741 via markerless replacement as follows. A fragment containing the *URA* locus was amplified from pRS416 using the oligos tp186 (5′CAGTAAAGTAAAGATCAGAACCAAAAATAGGCATATACACTTTTTATAGTGTACTGAGAGTGCACCATACCACAG) and tp187 (5′AGTAATCATGTAATATTGTAGTAAGGTTATGTATGTTCGTATGGTATGGGTGCGGTATTTCACACCGCATAGGG) which were designed to contain homology 288 bp 5′ and 28 bp 3′ of *RFU1* (underlined). The resulting PCR product was transformed into the yeast strain BY4741 and recombinants were selected on URA-plates. Surviving colonies were sequence-verified and designated TPY125 (RM/BY_RFU1). The RM11-1a *RFU1* ORF was amplified from genomic DNA using oligos tp148 (5′ AAGAAGAGTCAAGGAGTTGAG) and tp151 (5′ GTGTTACATCTTTTGGATCACC), transformed into TPY125, and selected on plates containing 1 mg/ml 5-fluoroorotic acid (Research Products International). Two independent transformants were sequence-verified and designated TPY127 (BY/RM_RFU1).

### Quantitative PCR

Yeast cultures in rich medium were grown overnight to saturation, back-diluted to an optical density of 0.06, and then grown for another 6-8 h in rich medium, until reaching an optical density (OD600) of 0.7. After freezing cell pellets in liquid nitrogen, RNA was isolated using Qiagen RNAeasy mini kit and cDNA was synthesized with Invitrogen Superscript III. Transcript levels of *RFU1* and *ACT1* were quantified with the ThermoFisher SYBR green assay following the manufacturer’s instructions, on each of two biological replicate cultures for the laboratory strain wild-type and two biological replicates of each of the two independent transformants of the *RFU1* allele-swap strain, with three technical replicates in each case.

### Nematode strains and culture methods

The following *C. elegans* strains were obtained from the *Caenorhabditis* Genetics Center (CGC, University of Minnesota) and cultured using standard solid media conditions (Ward *et al.* 1988): AM141 [*rmIs133(P(unc-54)*::*Q40*::*YFP)*] (Morley *et al.* 2002) and VC889 *wwp-1(gk372)*. *wwp-1(gk372)* harbors an 1509 bp deletion and an 149 bp insertion that removes the 5′UTR, start codon and first three exons of *wwp-1 (**Astin et al. 2008**)*. AM141 males were crossed to *wwp-1* mutant hermaphrodites to create GL400 [*wwp-1(gk372)*; *unc-54p*::*Q40*::*YFP(rmIs133)*].

### Microscopy and quantification in nematodes

Synchronous animal populations were obtained by two-hour egg lay. The synchronous populations were cultured at 15°, with daily transfer to fresh NGM plates beginning at the onset of egg laying. At each indicated developmental time point, animals were selected from the population to use for microscopy. Animals were paralyzed in 1 mM levamisole and mounted on 2% agarose pads with glass coverslips and immediately analyzed using a Zeiss Axio Imager A2 upright microscope and a 63x/1.4 objective. Animals were analyzed using 470/40 nm excitation and 525/50 nm emission wavelengths, and images were captured with 25 ms exposure using a Zeiss Axiocam 506 camera. Pixel density was analyzed with Image J. Puncta were quantified by manual count. Approximately 25 animals per condition were used, and three biological replicates of the experiment were performed.

### Cell culture, microscopy and image analysis of human cells

HEK293T cells (1x10^5^ per well) were seeded in the center 4 wells of 8-well collagen-coated coverglass slides (Lab-Tek II) containing a transfection mix of 17.8 pmol siRNA pool (NT#2 or NEDD4 siGENOME smartpools, Dharmacon), 260 ng pEGFP-C1-HTT(1-558) (Miller *et al.* 2012), 0.88 μl Lipofectamine 2000, 38 μl Opti-MEM (Gibco), and 135 μl DMEM containing 10% FBS. Media was changed every day, and cells were analyzed by confocal microscopy at 48 h post-transfection. Live HEK293T cells were imaged in the presence of Hoechst 33342 (5 µg/ml) chromatin stain, in a medium consisting of (in mM) 120 NaCl, 3.5 KCl, 1.8 CaCl_2_, 1 MgCl_2_, 0.4 KH_2_PO_4_, 5 NaHCO_3_, 1.2 Na_2_SO_4_, 20 TES, 25 glucose using a Zeiss LSM780 laser scanning confocal microscope with a Plan-Apochromat 63×/1.4 oil lens and standard spectral configuration for Hoechst 33342 and GFP. 3×3 tiled z-stacks of 14 planes with 2 µm spacing and 0.26 µm/pixel resolution were recorded at evenly monolayer parts of the culture selected under bright field only observation. Recorded z-stacks were maximal intensity projected and segmented for nuclei and for aggregates using Image Analyst MKII, using the same pipeline for all recordings. Aggregate size was analyzed by binning them categories of smaller or larger than 50 µm^2^ area. Four biological replicates were measured for each condition (*i.e.*, siNT and siNEDD4) and 160 aggregates were measured in each replicate, for a total of 1280 aggregates analyzed. Histogram and statistical Mann-Whitney rank sum test of aggregate size was performed in Mathematica 10 (Wolfram Research, Champaign, IL). Western blots of NEDD4, Q144-HTT-1-558-GFP, and HSP60 (loading control) were carried out as described (Miller *et al.* 2012) using antibodies ab236512, ab13970, and ab46798, respectively (Abcam, Cambridge, UK).

### Statement on Data Availability

Strains and plasmids are available upon request. Supplemental files available at FigShare. Table S1 contains strains used in this study. Table S2 gives linkage signals at genomic marker locations. Table S3 shows linkage between RFU1 genotype and RFU1 expression. Supplemental material available at Figshare: https://doi.org/10.25387/g3.6085481.

## Results

### A quantitative fluorescence microscopy assay for protein aggregation in yeast

To identify loci at which naturally occurring polymorphisms govern protein aggregation in yeast, we made use of a panel of meiotic segregants derived from the cross of a standard laboratory yeast strain (BY4716) and a vineyard isolate (RM11-1a) (Brem and Kruglyak 2005). To model protein aggregation in these strains, we harnessed an expanded allele of the first exon of the human huntingtin gene (encoding a 75 glutamine tract) fused to GFP (Hughes *et al.* 2001). Prior studies have shown that constitutive expression of this GFP-fused expanded huntingtin fragment (hereafter referred to as Htt-Q75-GFP) in yeast results in the formation of protein aggregates discernable as fluorescent GFP foci in the cytoplasm (Duennwald *et al.* 2006a). Aggregation of this protein has no obvious toxicity on the yeast cell and was found to rely upon the prion state of the Rnq1 protein, whereby [*PIN+*] cells had high levels and [*pin*-] cells had low levels of protein aggregation (Duennwald *et al.* 2006a; Hughes *et al.* 2001).

**Figure 1:**
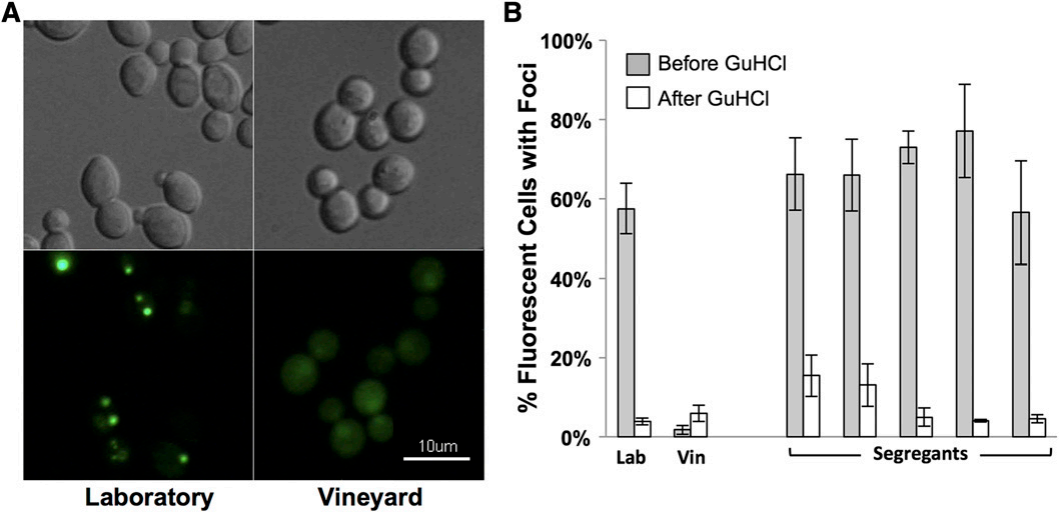
Rnq1 prion modifies Htt-Q75-GFP protein aggregation. (A) Laboratory and vineyard parent strains induced to express the Rnq1-YFP fusion protein for 12 h were imaged. Representative images of DIC (top) and fluorescent patterns of the Rnq1-YFP protein (bottom) are shown. Focal YFP signal present in the Laboratory strain images indicate [*PIN+]* cells while diffuse YFP signal in the vineyard strain indicates *[pin-]* cells. (B) Aggregation of the Htt-Q75-GFP protein was quantitated in Laboratory (Lab), Vineyard (Vin) parent strains and five arbitrarily selected segregants before and after successive passaging on GuHCl plates to eliminate endogenous prions. Aggregation is represented as the percentage of GFP-fluorescent cells with foci.

Our initial investigations revealed that the laboratory parent strain and all segregant strains had significant aggregation of the Htt-Q75-GFP (Figure 1). Conversely, the vineyard parent strain had relatively low levels of protein aggregates. As aggregation of the Htt-Q75-GFP protein has been shown to be influenced by the [*PIN+*] prion, we tested the prion state of the parent strains using an Rnq1-YFP reporter assay (Liu and Lindquist 1999). This analysis revealed that the laboratory strain, but not the vineyard strain, was [*PIN+*] (Figure 1A). Successive passages of both parent strains and all segregants on plates containing guanidine hydrochloride cured strains of the Rnq1 prion, and dramatically reduced aggregation of the Htt-Q75-GFP protein in the laboratory parent and all segregants (Figure 1B).

**Figure 2:**
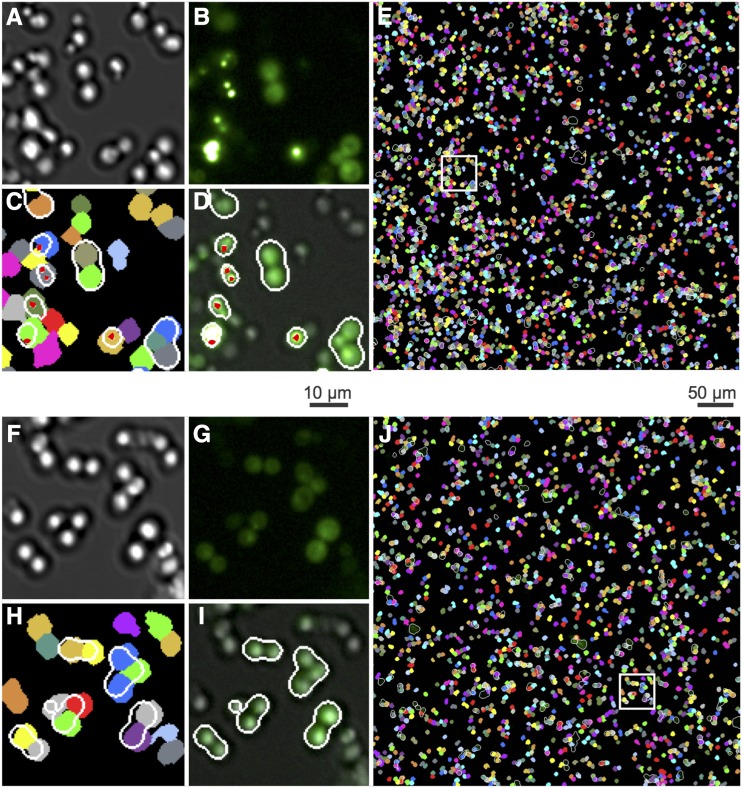
Quantitative imaging methods for protein aggregation in yeast. Automated protein aggregate counting in yeast attached to glass-bottomed 96-well plates. Representative fields are shown for a segregant exhibiting high aggregation (Upper Panel, A-E) and a segregant with low aggregation (Lower Panel, F-J). Captured DIC (A, F) and fluorescent (B, G) images were used to digitally segment cells and fluorescent patterns respectively. Automated Image processing segmented DIC images to identify individual cells (C, H colored segments) and binarized GFP fluorescence after high pass or low band pass spatial filtering to mark protein aggregates (red dots) or diffuse fluorescence (outlined in white), respectively (D, I). Thus, D,I images represent GFP and DIC overlaid images with cells exhibiting fluorescence circled in white trace, and GFP-positive foci marked with a red dot. These data were then used to calculate the number of individual cells that also contain foci and diffuse fluorescence features and subsequently used to determine the percentage of fluorescent cells with foci. The white boxes in (E, J) refer to regions magnified in the corresponding smaller four images to the left.

**Figure 3:**
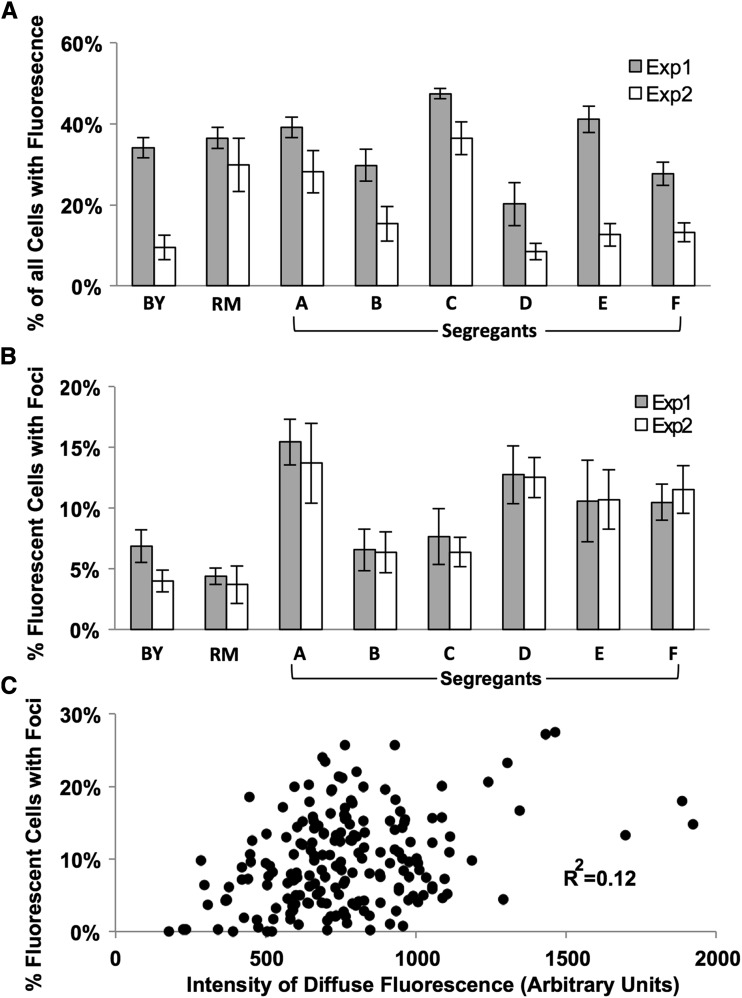
A quantitative fluorescence microscopy assay for Htt-Q75-GFP aggregation. Fluorescence patterns were quantitated in parent strains and segregants using an empirically derived algorithm to identify cells with fluorescence (A) or the percentage of fluorescent cells with foci (B) in two distinct experiments. (C) The diffuse fluorescence intensity was quantitated in all replicates of parent strains and 29 segregant strains in an area of the cell excluding foci. This intensity was plotted against the percentage of fluorescent cells with foci in the same samples. Each strain had at least five independent replicates quantitated; each point represents an average of over 1000 cells analyzed per replicate.

We used these prion-cured strains to develop a quantitative microscopy method to assay Htt-Q75-GFP aggregate formation, including computational strategies to recognize cells and aggregates in microscopy images (Figure 2). While the absolute level of fluorescent cells in a given strain differed between experiments, the percentage of fluorescent cells with foci was reproducible between experiments (Figure 3A,B). Using the latter parameter also allowed us to standardize for variation in the percentage of cells with fluorescence and thus for effects of inherent Htt-Q75-GFP expression differences upon aggregation levels. Our analysis found low correlation between the percentage of fluorescent cells with foci and the intensity of diffuse fluorescence, suggesting that expression differences among the segregants had a minimal effect on the propensity of a strain to form Htt-Q75-GFP protein aggregates (Figure 3C).

### Htt-Q75-GFP aggregation is a complex trait in yeast

A panel of 29 prion-cured meiotic yeast segregants and parent strains constitutively expressing the Htt-Q75-GFP protein was assayed for the propensity to form protein aggregates. Relative aggregation (defined as the percent of fluorescent cells with foci) was similar in the two parent strains and varied over 10-fold among the segregants. The percent of fluorescent cells with foci among the 29 segregants ranged from 0.2 to 21.9%, with these extremes representing values threefold higher and 20-fold lower than those detected in the parent strains (data not shown). This broad range suggested transgressive segregation of a complex genetic network regulating Htt-Q75-GFP protein aggregation, with a given parent harboring pro-aggregation alleles at some loci and anti-aggregation alleles at others.

**Figure 4:**
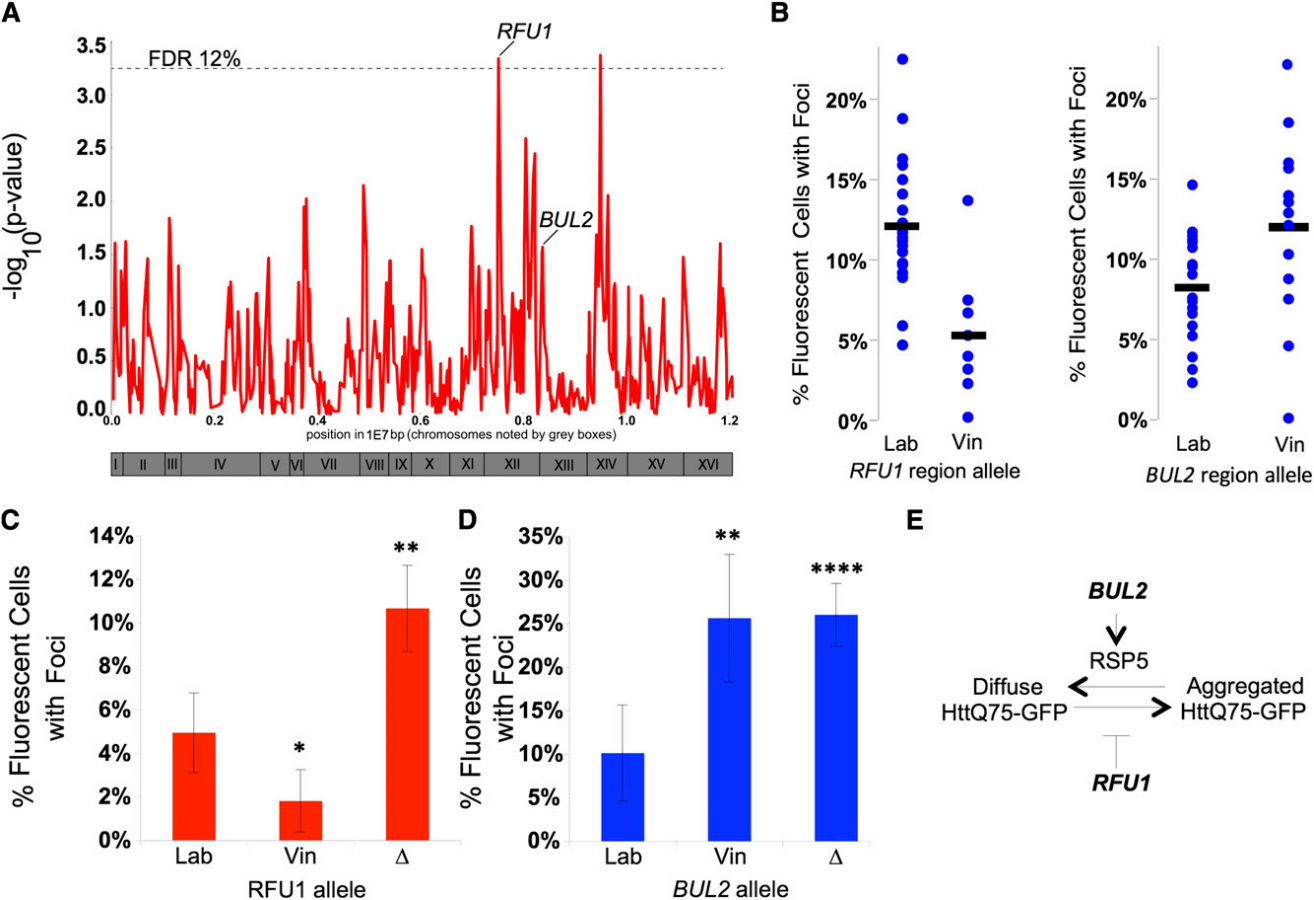
QTL analysis identifies segregating loci and genes that influence Htt-Q75-GFP aggregation. (A) Strength of linkage across segregant strains relative to genomic position. Dotted line represents the signal strength corresponding to a false discovery rate of 12% in randomly permuted data. Loci with validated genetic modifiers are labeled. Roman numerals refer to relative positioning on the yeast chromosomes. (B) Aggregation of segregant strains (blue dots) binned by allele at the *RFU1* locus (left) or *BUL2* locus (right). (C, D) Aggregation of the Htt-Q75-GFP protein was quantitated in the laboratory strain carrying either the laboratory allele (Lab), vineyard allele (Vin), or deletion cassette (∆) of the (C) BUL2 gene or (D) RFU1 gene. (E) Model for the influence of BUL2 and RFU1 on aggregation of the Htt-Q75-GFP protein. * = *p*-value < 0.05; ** = *p*-value < 0.01; *** = *p*-value < 0.005 in tests of the indicated strain relative to the laboratory wild-type. Error bars report standard error of the mean.

To map loci underlying variation in protein aggregation in this cross, we carried out linkage mapping using previously reported genotype data for each segregant in turn at each of 1244 DNA sequence markers (Brem and Kruglyak 2005). The mapping analysis found several markers where parental inheritance was linked to Htt-Q75-GFP protein aggregation trait. Two genetic loci exhibiting the highest linkage mapped at a false discovery rate of 12% (Figure 4A, Table S2). These peaks explain 38% of the phenotypic variance independently. For both of these loci, the laboratory parent allele is associated with higher aggregation (data not shown). We built a model incorporating these top two loci and it accounts for 54% of the variance. We considered these two mapped loci as prime candidates for novel determinants of cytoplasmic protein aggregation control.

### RFU1 and BUL2 are novel modifiers of Htt-Q75-GFP aggregation in yeast

As a first test to validate genes near our linking markers that were causal for changes in Htt-Q75-GFP aggregation across strains, we focused on *RFU1*, which was immediately adjacent to a linking marker on chromosome XII (Figure 4A,B). *RFU1* encodes a protein that inhibits the Doa4 deubiquitinase at the late endosome preventing recycling of ubiquitin in the cell (Kimura *et al.* 2009). Doa4 de-ubiquitinates the integral membrane proteins in the late endosome, recycling ubiquitin before the substrate is targeted for transport to the vacuole (Amerik *et al.* 2000; Nikko and Andre 2007). Doa4 targets include several proteins that are ubiquitinated by the Rsp5 E3-ubiquitin ligase complex (Amerik *et al.* 2000; Nikko and Andre 2007; Kimura *et al.* 2009). Rsp5 has also been shown to have ligase activity on proteins in the cytosol. Upon heat stress, Rsp5 targets cytosolic misfolded proteins for ubiquitination and degradation, while also regulating polyQ protein aggregation turnover via autophagy in yeast (Fang *et al.* 2014; Lu *et al.* 2014). Given these critical roles of Rsp5 in response to proteomic stress and its functional and genetic relationship to RfuI we reasoned that a polymorphism at the *RFU1* locus might influence Htt-Q75-GFP aggregation.

To test this, we constructed strains in an isogenic background differing only in the allele they carried at *RFU1* and assayed these strains for aggregation of the Htt-Q75-GFP protein. The results revealed that cultures of strains bearing the vineyard *RFU1* allele had a reduction of >50% in cells exhibiting aggregates as compared to the isogenic laboratory parent while a *RFU1* null mutation was sufficient for an increase of >twofold in Htt-Q75-GFP aggregation (Figure 4C). These results make clear that RFU1 serves as a modifier of protein aggregation, and that the vineyard strain harbors a genetic variant that results in less aggregation than the laboratory strain. Sequencing revealed no protein-coding changes between these two strains with in the *RFU1* coding region in either the parent genome sequences or in our allele-swap strains. However, the vineyard strain’s allele at the *RFU1* locus was linked with lower expression of the *RFU1* transcript among the progeny of the cross between these two parents (Brem and Kruglyak 2005); Figure S1), and the vineyard allele was sufficient for low expression of *RFU1* in our isogenic allele-swap experiment (Figure S2), suggesting that *cis*-acting polymorphisms between the strains at the *RFU1* locus modulate its expression.

As Rfu1 modifies ubiquitination of Rsp5 targets (and is in turn regulated by Rsp5), and Rsp5 is a regulator of cytosolic misfolded protein aggregation (Fang *et al.* 2014; Lu *et al.* 2014), we reasoned that other genes that affect ubiquitin regulation may also influence aggregation in our screen. Searching other loci that segregate with protein aggregation, we found a marker on chromosome XIII adjacent to the gene *BUL2*, an arrestin-related adapter protein of the Rsp5 E3-ubiquitin ligase complex. Bul2 mediates binding of the Rsp5-ubiquitin ligase complex to target membrane-bound proteins (Helliwell *et al.* 2001; Novoselova *et al.* 2012). Thus far, a role for Bul2 in the ubiquitination of cytosolic proteins has not been noted. A single nucleotide polymorphism in *BUL2* between the vineyard and laboratory parent strains has been described that results in reduced activity of the laboratory strain allele compromising the ability of Bul2 to efficiently recycle the Gap1 amino acid permease from the membrane (Kwan *et al.* 2011). We hypothesized that this variation in the *BUL2* gene also influences cytoplasmic Htt aggregation.

Although the *BUL2* locus does not significantly segregate with our phenotype in our linkage analysis (*p*-value = 0.027, Figure 4B, right), given the strength of the literature on its interaction with Rsp5, we decided to test how the *BUL2* polymorphism affects Htt-Q75-GFP aggregation. We compared aggregation of the Htt-Q75-GFP protein in strains carrying different alleles of the *BUL2* locus in an otherwise isogenic background (Kwan *et al.* 2011). The results revealed robust and significant effects of *BUL2* genotype on Htt aggregation: strains carrying the vineyard *BUL2* allele had 2.5-fold more cells with aggregates than isogenic strains carrying the laboratory allele (Figure 4D). A similar 2.6-fold increase in protein aggregation was also seen in isogenic *bul2∆* cells (Figure 4D). These results confirm *BUL2* as a novel modifier of cytoplasmic protein aggregation and indicate that the vineyard allele of *BUL2* behaves as a loss-of-function allele with respect to aggregation suppression.

Taken together, our data indicate that both *RFU1* and *BUL2* activity suppress cytoplasmic Htt-Q75-GFP aggregation (Figure 4 C,D). These data are consistent with the known role for the Rsp5 ubiquitin ligase in polyQ aggregation control (Lu *et al.* 2014). Given that Bul2 activates Rsp5 and Rfu1 inhibits the deubiquitinase function of Doa4, it would stand to reason that lowering activities of either of these components could lead to higher aggregation as we see in our study (Figures 4E). These data indicate that in addition to Rsp5, interacting partners may be responsible for modulating polyQ aggregation in yeast, though the specific mechanism by which this network does so needs to be further clarified.

### The E3-ubiquitin ligase gene wwp-1 regulates polyglutamine aggregation in C. elegans

In order to determine whether the effect of this E3-ubiquitin ligase on polyQ aggregation was conserved in higher eukaryotes we used an assay similar to our yeast approach in a transgenic *C. elegans* model. Expression of polyQ-YFP protein from the muscle-specific *unc-54* promoter in *C. elegans* has been shown to lead to substantial aggregation within the body wall muscle cells of the animal upon reaching adulthood (Morley *et al.* 2002). Using this system, we quantified aggregation of polyQ-YFP in wild-type animals and in two strains, each harboring distinct loss of function alleles of the Rsp5 homolog *wwp-1*. On the basis of the role in polyQ aggregation of Rsp5 and its partners, we predicted that *wwp-1* null worms would have higher levels of polyQ-YFP aggregation than animals that are wild-type at the *wwp-1* locus.

Aggregation of the polyQ-YFP reporter was measured in *wwp-1* loss of function strains and wild-type controls. We found increased YFP puncta at larval and adult time points in both *wwp-1(gk372)* and *wwp-1(ok1102)* animals, as compared to controls (Figure S3). Animals harboring the *wwp-1(ok1102)* allele result in many dead embryos; because of this, we pursued time course experiments with only the *wwp-1(gk372)* strain.

**Figure 5:**
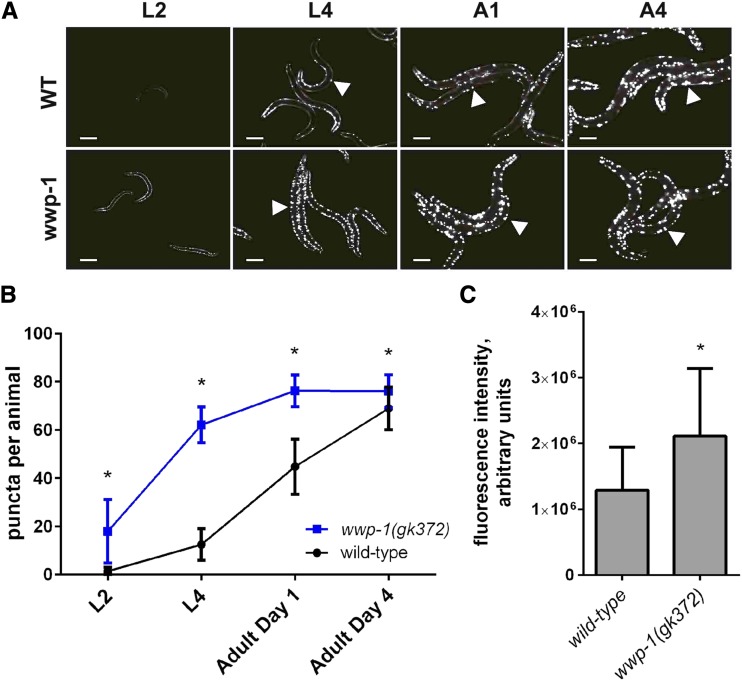
WWP-1 suppresses aggregation in *C. elegans*. (A) Representative images of wild-type or isogenic *wwp-1 null (wwp-1 gk372)* animals expressing unc-54p::Q40::YFP at both larval (L2, L4) and adult (A2, A4) development stages. All images are at 20X; white bar represents 200 µm. Arrowheads indicate YFP-puncta. (B) Time course quantification of YFP puncta per animal in wild-type or *wwp-1* animals expressing unc-54p::Q40::YFP. YFP puncta were quantified from images of a minimum of 70 animals of each genotype, per developmental time point. (C) Quantitation of whole worm fluorescence intensity in wild-type (n = 81) or *wwp-1* (*n* = 76) animals at L2 development stage and represented relative to wild-type levels. Error bars indicate standard error. *= *p*-value < 0.0001, one-way ANOVA, Sidak’s multiple comparisons test.

We tested polyQ::YFP aggregation in *wwp-1(gk372)* null and wild-type strains of *C. elegans* at four developmental timepoints: larval stages L2 and L4, and days 1 and 4 of adulthood. We found that *wwp-1* null mutants accumulated aggregates more rapidly throughout life compared to wild-type and thus had more puncta per animal at all timepoints measured. Very few polyQ-YFP puncta were observed in wild-type juvenile animals, whereas *wwp-1* mutants had on average 17 puncta per animal at the L2 stage (Figure 5A,B). Aggregation peaked in wild-type worms at day 4 of adulthood with 68.9 puncta per worm; by contrast, *wwp-1* nulls reached 76.1 puncta per worm by day 1 of adulthood and maintained it through the end of the study (Figure 5B). In addition to increased puncta, we found that juvenile *wwp-1(gk372)* mutants had higher levels of YFP fluorescence at early stages of development, largely before puncta were observed (Figure 5A, C), suggesting that *wwp-1* mutants had higher steady-state levels of the polyQ-YFP protein. These data dovetail with our discovery genetic variation in genes that modulate E3-ubiquitin ligase activity can suppress protein aggregation in yeast, and highlight *wwp-1* as a determinant of steady-state levels and aggregation of the polyQ protein.

### The E3-ubiquitin ligase NEDD4 regulates polyglutamine aggregation in human cells

**Figure 6:**
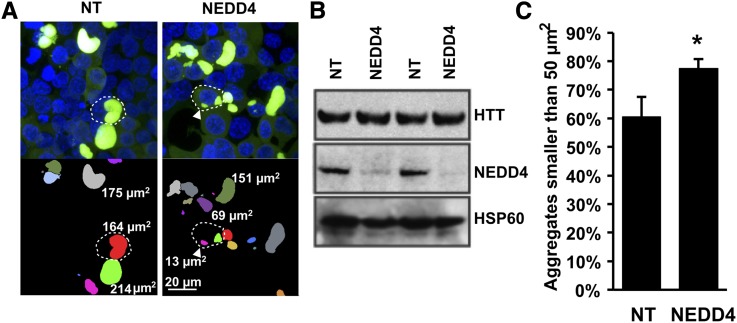
NEDD4 knock-down causes a decrease in aggregate size. (A) Representative images of HEK293T cells expressing Q144-HTT-1-558-GFP 48 h post transfection and stained with Hoechst dye with either control siNT (non-targeting; left), or siRNA against NEDD4 (right). Fluorescent images are shown in upper panels and segmented images denoted with aggregate areas are shown in lower panels. Dotted white lines indicate representative cells, diffusely stained cells are present directly below the cell with a dotted outline in the top right NEDD4 panel. (B) Representative immunoblots showing siRNA knock-down of NEDD4 *vs.* non-targeting siRNA controls (NT). HSP60 expression was unchanged. (C) Percentage of aggregates smaller than 50 µm^2^ in cells. Error bars report standard error (n = 4). The difference between treatments was significant *, significance at *P* < 0.05 by Mann-Whitney rank sum test.

The human ortholog of Rsp5, NEDD4, has been previously implicated in aggregation of membrane-associated proteins (Tardiff *et al.* 2013; Tofaris *et al.* 2011). Given the impact on cytoplasmic mutant huntingtin aggregation of Rsp5-interacting genes in yeast and the Rsp5 ortholog *wwp-1* in nematodes, we hypothesized that NEDD4 also has a role in regulating cytoplasmic polyQ aggregation in mammalian cells. To test this, we cultured HEK293T cells expressing an aggregation-prone HTT construct, Q144-HTT-1-558-GFP, which readily forms cytoplasmic polyglutamine-GFP aggregates (Miller *et al.* 2012). We used quantitative image analysis to quantify the number and morphology of polyQ-GFP aggregates in HEK293T cells after 48 hr of polyglutamine expression, in cells transfected with siRNA for knockdown of NEDD4 and in non-targeting siRNA controls (Figure 6A-C). The results revealed changes in aggregate morphology (Figure 6C). Quantification of the size of polyQ aggregates revealed a significant increase in the relative number of small fluorescent foci in NEDD4 knockdown cells (Figure 6C). These observations are consistent with a role for NEDD4 in regulating polyglutamine aggregation in human cells.

## Discussion

In this work, we have used natural variation between two yeast strains to map two loci that modify aggregation of an expanded huntingtin exon-1 protein. We then used molecular methods to identify novel regulation of polyQ aggregation by two mapped yeast genes, *BUL2* and *RFU1*, and in each case we established the impact of natural genetic variants on the aggregation phenotype. Neither of these mutants have been shown in previous work to affect protein aggregation or associated phenotypes (Giorgini *et al.* 2005; Willingham *et al.* 2003). Our efforts to deconvolve protein aggregation from cellular toxicity by using a non-toxic expanded huntingtin construct and curing the cell of the *[PIN+]* prion put us in the unique position to identify novel modifiers of protein aggregation (Duennwald *et al.* 2006a; Duennwald *et al.* 2006b; Giorgini *et al.* 2005; Willingham *et al.* 2003).

Much of the literature to date characterizing the α-arrestin Rsp5 adapter protein Bul2, and Rfu1, an inhibitor of the Doa4 deubiquitinase, has focused on the regulation and recycling of ubiquitinated membrane-bound proteins. Bul2 promotes ubiquitination by binding a given target and the Rsp5 E3-ubiquitin ligase facilitating ubiquitination of the target (Helliwell *et al.* 2001; Novoselova *et al.* 2012). Rfu1 prevents deubiquitination of the Rsp5-ubiquitinated targets by reducing activity of the Doa4 deubiquitinase (Kimura *et al.* 2009). In addition, Rsp5 contributes to degradation of Rfu1 when protein homeostasis is challenged by elevated temperatures (Kimura *et al.* 2014; Kimura *et al.* 2015). From these reports it is clear that Rsp5, Rfu1, and Bul2 act within a larger network to regulate ubiquitination, ubiquitin recycling, and protein homeostasis of membrane bound proteins at the cell membrane and the late endosome.

In addition to its function at the cell surface, recent reports outline the importance of Rsp5 in modifying protein aggregation in the cytosol. The Rsp5 E3 ubiquitin ligase targets cytosolic misfolded proteins, promotes accumulation and turnover of expanded huntingtin via autophagy and relieves toxicity of the aggregation prone α-synuclein protein in yeast (Fang *et al.* 2014) (Lu *et al.* 2014) (Tardiff *et al.* 2013). In mammalian cells, NEDD4 (the human ortholog of the yeast *RSP5*) ubiquitinates and regulates turn-over of a mutant form of the membrane-associated protein α-synuclein. This is consistent with our previous detection of NEDD4 as a modifier of mutant huntingtin cytotoxicity in human cells in which knockdown of NEDD4 decreased huntingtin-mediated cell death by 30% relative to controls (Miller *et al.* 2012). The current work goes further to show that NEDD4 knockdown modifies protein aggregation by increasing the level of smaller protein aggregates that can be seen via fluorescence microscopy.

Our current work not only supports the idea that RSP5*/*NEDD4 is important to maintenance of protein homeostasis but further implicates the broader ubiquitin regulation network as playing a role in this process. We show that Rfu1 and Bul2 both modify protein aggregation in the cytosol of the aggregation-prone mutant huntingtin exon 1. Given the complex nature of protein aggregation, it is likely that additional genes within this network also play a role in this phenotype. Demonstrating this, the two parent strains aggregate the protein at a similar frequency, yet segregants exhibit a large diversity in their propensity to aggregate the Htt-Q75-GFP protein. It is highly possible that additional modifiers of this phenotype have been mapped in this study, most likely in the uncharacterized locus on chromosome XIV, though no obvious candidate is present in this locus (Table S3). Other mapped loci with lower confidence may also harbor modifiers. For instance, a locus containing *ECM21* on chromosome II shows linkage to protein aggregation (*p*-value = 0.024) at a similar level as the experimentally-verified *BUL2* locus (*p*-value = 0.027). *ECM21* codes for another arrestin-related protein that, like Bul2, serves as an adapter protein for Rsp5-mediated ubiquitination of membrane proteins at the cell surface and associated endosomes (Lin *et al.* 2008). This may represent another component in the ubiquitin-modifying network that influences this aggregation phenotype. Future work will establish how polymorphisms in this and other candidate loci modulate activity of the ubiquitination network for proteostatic control in the cytosol.

Our discovery of Rsp5 interaction partners as new determinants of polyQ aggregation in yeast led us to the study of the Rsp5 ortholog in *C. elegans*, *wwp-1* (Huang *et al.* 2000). Previous RNAi-based screens in the worm have failed to identify *wwp-1* as a modifier of protein aggregation (Nollen *et al.* 2004; Silva *et al.* 2011; Van Ham *et al.* 2008). The observation of robust and reproducible increases in age-dependent aggregation in a mutant of *wwp-1* suggests that partial RNAi knockdown and genetic manipulation of this locus may result in quite distinct phenotypes. Interestingly, *wwp-1* is required for lifespan extension in worms under dietary restriction (DR) (Carrano *et al.* 2009), suggesting a model where suppression of protein misfolding is critical for lifespan extension under DR. Such a model would be consistent with the known benefits of DR in worms expressing polyQ or amyloid beta and underline the relationship between proteostasis maintenance and healthy aging (Steinkraus *et al.* 2008).

Our results in yeast suggest that a decreased function of Rsp5 complex may result from a *BUL2* knockout which subsequently increases aggregation (Figure 4C). In worms, the *wwp-1* mutants have more aggregation and higher levels of steady state protein as measured by fluorescence (Figure 5B). In HEK293T cells, we show that decreased NEDD4 results in an increase in the number of smaller aggregates (Figure 6). Regardless of disparate aggregation phenotypes, our work shows that disruption of a homologous E3 ubiquitin ligase in divergent species interferes with aggregation of a polyglutamine protein supporting the idea that this network is critical to the maintenance of proteostasis. However, the discrepancy in steady-state levels of aggregated proteins and their visualization via fluorescent tag are a summation of many interacting processes. Altering one component of a single step in this network may manifest itself differently in yeast than in a mammalian model due to evolved genetic networks controlling these phenotypes in higher eukaryotes. For instance, humans have 28 E3-HECT ubiquitin ligases while nematodes and yeast have only seven and five E3-HECT ubiquitin ligases, respectively (Finley *et al.* 2012; Scheffner and Kumar 2014). This demonstrates the potential for a much broader network influencing a phenotype in mammalian cells than in either nematodes or yeast. Ultimately, further characterization is needed to understand the differences in the aggregation-modifying networks surrounding the homologs of NEDD4 in mammalian cells.

The emerging picture from this work and that of others is one in which Rsp5/WWP-1/NEDD4 complex functions as one modulator in a web of regulators controlling protein homeostasis of cytoplasmic misfolded proteins, likely through the modulation of the ubiquitination network. Therefore, the Rsp5 complex, and those proteins involved in its activity, are an attractive target for novel drugs to treat a range of proteinopathy disorders. Given the complex nature of the protein aggregation phenotype, we anticipate that additional candidate genes at the loci mapped in our yeast screen will serve as the basis for future molecular validation efforts in invertebrates and mammals and may provide new candidate targets for therapeutic development.
